# Supplementary material for: Effects of a Combined High-Intensity Interval Training and Resistance Training Program in Patients Awaiting Bariatric Surgery: A Pilot Study
Source: Sports (Basel). 2019 Mar 25;7(3):72. doi: 10.3390/sports7030072 (PMC6473567; doi:10.3390/sports7030072)
Supplement: Supplementary file 1 [file sports-07-00072-s001.pdf]

SUPPLEMENTARY MATERIAL

Appendix I. Changes (%) in all the variables evaluated. Qualitative inference and likelihood (%) of the effects being positive/trivial/negative for each of the groups.

	EG (n=3)					CG (n=3)				
**Variables**	**% Change (90% CL)**	**Chances that the true effects were substantial**				**% Change (90% CL)**	**Chances that the true effects were substantial**			
		**Harmful**	**Trivial**	**Beneficial**	**Qualitative inference**		**Harmful**	**Trivial**	**Beneficial**	**Qualitative inference**
**BMI (kg·m^{-2})**	9.0 (3.0;14.6)	2	1	97	Unclear	-0.5 (-3.0;1.9)	48	36	16	**Possibly harmful**
Weight (kg)	9.0 (3.0;14.6)	1	4	95	Unclear	-0.5 (-3.0;1.9)	4	94	2	Likely trivial
EBW (kg)	26.9 (8.7;41.5)	2	1	97	Unclear	-1.4 (-8.2;4.9)	36	52	12	Possibly harmful
**EBW (%)**	19.7 (5.7;31.7)	3	0	97	Unclear	-0.9 (-5.1;3.1)	48	37	15	**Possibly harmful**
Fat mass (kg)	17.5 (-7.4;36.6)	6	7	87	Unclear	0.7 (-1.9;3.3)	1	96	2	Very likely trivial
**Fat mass (%)**	9.3 (-11.0;25.9)	12	7	82	**Unclear**	1.2 (0.6;1.9)	0	93	7	Likely trivial
FFM (Kg)	-3.7 (-8.1;0.9)	76	21	3	Unclear	1.7 (-0.5;4.0)	2	76	22	Likely trivial
**FFM (%)**	5.8 (-4.0;16.6)	8	10	82	**Likely beneficial**	1.2 (0.7;1.7)	0	96	4	Very likely trivial
Waist (cm)	5.0 (5.0;13.6)	7	20	73	Unclear	-0.4 (-3.0;2.2)	6	91	4	Likely trivial
Hip (cm)	6.5 (1.1;11.6)	2	4	93	Unclear	0.3 (-1.4;1.9)	3	92	5	Likely trivial
WHR (cm)	-1.6 (-9.2;5.5)	29	62	10	**Possibly harmful**	-0.7 (-1.7;0.3)	1	99	0	Very likely trivial
**Visceral fat (%)**	15.9 (-6.8;33.8)	5	9	86	**Unclear**	2.3 (-4.5;8.6)	4	82	14	Likely trivial
SBP (mmHg)	144.22±37.39	27	14	59	Unclear	-0.8 (-14.8;11.6)	45	20	34	Unclear
DBP (mmHg)	83.22±18.68	10	21	68	Unclear	4.6 (-15.0;20.9)	19	17	63	Unclear
HR_{rest} (bpm)	56.33±12.34	68	29	2	**Possibly harmful**	-1.7 (-13.3;8.7)	12	82	6	Likely trivial
$VO_{2peak\ abs}$ (L·min^{-1})	2.62±1.11	20	77	3	Likely trivial	6.1 (-21.3;43.0)	19	30	51	**Possibly beneficial**
$VO_{2peak\ abs/FFM}$ (mL*FFM^{-1}·min^{-1})	39.92±8.01	5	18	76	**Likely beneficial**	4.4 (-21.1;38.0)	25	23	52	**Possibly beneficial**
MDS_{D-Q} (N·m)	151.84±36.10	19	70	11	Possibly trivial	4.0 (-4.3;13.0)	4	64	32	**Possibly beneficial**
MDS_{D-H} (N·m)	86.53±24.87	21	63	16	Possibly trivial	-8.6 (-19.7;4.0)	36	62	2	Unclear
MDS_{ND-Q} (N·m)	149.88±47.94	1	8	91	**Likely beneficial**	0.4 (-8.5;10.3)	15	66	19	Possibly trivial
MDS_{ND-H} (N·m)	75.81±14.93	5	10	85	**Likely beneficial**	6.7 (1.3;12.3)	1	14	84	**Likely beneficial**
MIS_{D-Q} (N·m)	141.90±19.18	12	85	4	Likely trivial	-5.6 (-22.1;14.2)	55	33	12	Unclear
MIS_{D-H} (N·m)	95.83±23.39	35	52	12	Unclear	-13.5 (-37.2;19.2)	70	21	9	Unclear
**MIS_{ND-Q} (N·m)**	147.50±3.47	13	53	34	**Possibly beneficial**	-9.0 (-19.1;2.3)	82	14	4	Unclear
MIS_{ND-H} (N·m)	90.30±15.95	11	81	8	Likely trivial	-1.4 (-31.3;21.7)	20	64	16	Possibly trivial

PAP = Physical Activity Program; EG = experimental group; CL = coefficient limits; BMI = body mass index; EBW = excess body weight; FFM = fat free mass; WHR = waist-to-hip ratio; SBP = systolic blood pressure; DBP = diastolic blood pressure; HR_{rest} = heart rate at rest; $VO_{2peak\ abs}$ = absolute peak oxygen uptake; $VO_{2peak\ abs/FFM}$ = absolute peak oxygen uptake normalized to FFM; MDS = maximal dynamic strength; D = dominant; ND = non-dominant; Q = quadriceps; H = hamstrings; MIS = maximal isokinetic strength.
